# Aberrant Feeding and Growth in Neonates With Prenatal Opioid Exposure: Evidence of Neuromodulation and Behavioral Changes

**DOI:** 10.3389/fped.2021.805763

**Published:** 2022-01-21

**Authors:** Elizabeth Yen, Jill L. Maron

**Affiliations:** ^1^Mother Infant Research Institute, Tufts Medical Center, Boston, MA, United States; ^2^Department of Pediatrics, Tufts University School of Medicine, Boston, MA, United States; ^3^Department of Pediatrics, Women & Infants Hospital of Rhode Island, Providence, RI, United States; ^4^Warren Alpert Medical School of Brown University, Providence, RI, United States

**Keywords:** opioid epidemic, Neonatal Abstinence Syndrome, Inflammation, Neuromodulation, Feeding dysregulation, metabolic syndrome

## Abstract

Opioid use disorder (OUD) among pregnant women over the last decade has led to more than a fivefold increase in the number of neonates born with withdrawal signs known as Neonatal Abstinence Syndrome (NAS) or Neonatal Opioid Withdrawal Syndrome (NOWS). The impact of prenatal opioid exposure on these neonates remains a public health and research priority due to both its short and long-term effects on offspring. Among the adverse long-term effects associated with OUD is a metabolic syndrome with accompanying cardiovascular comorbidities. The susceptibility to metabolic diseases may begin as early as conception. Neonates born in a setting of prenatal opioid exposure are known to have aberrant early growth, e.g., lower birth weight and smaller head size, and dysregulated feeding behavior that ranges from feeding difficulty to hyperphagia which may predispose these neonates to metabolic syndrome in adulthood. However, studies on this topic are lacking. In this article, we describe the reported association between OUD and metabolic syndrome in adults, animal data linking opioid receptors with the development of diet-induced obesity, the inflammatory modulation of opioids and finally, neonatal salivary transcriptomic data from our laboratory that highlighted the sex-specific impact of opioids on the hypothalamic and reward receptors that regulate feeding behavior in opioid-exposed neonates. There is a great need for future research linking opioids with epigenetic and gene expression changes, as well as neuromodulatory effects in the developing brain, that may underlie the dysregulated feeding, growth, and long-term metabolic and cardiovascular risks for these neonates.

## Introduction

The rate of pregnant women misusing opioids has quadrupled between 1999 and 2014 (1), leading to a fivefold increase in Neonatal Abstinence Syndrome (NAS) or Neonatal Opioid Withdrawal Syndrome (NOWS) in the last decade (2). NAS affects multiple organs, including the gastrointestinal system, often resulting in uncoordinated feeding, excessive suck and hyperphagia, feeding intolerance, diarrhea, and poor growth (3, 4). The impact of these aberrant feeding and growth patterns is not well understood, yet they likely contributes to obesity or metabolic syndrome. Opioid use disorder (OUD) is associated with metabolic syndrome, defined as dyslipidemia, insulin resistance, obesity, and hypertension, predisposing these adults to cardiovascular disease, diabetes mellitus (DM), and death (5). Rodents exposed to opioids develop drug-induced obesity and DM type 2 (6), while administration of opioid antagonists reduces opioid craving and appetite for palatable food leading to weight loss (7, 8). The exact mechanisms by which drug addiction and metabolic syndrome are linked together are of growing interest, with some reporting no direct association (9), while others reporting disrupted reward circuitry, i.e., dopamine receptors, as a common denominator between opioid addiction, food dependence and obesity (10–12). To close this knowledge gap, our laboratory examines the expression of hypothalamic and reward genes that regulate feeding behavior in opioid-exposed neonates, with evidence of abnormal reward signaling and behavioral changes that may predispose these neonates to long-term growth and metabolic issues (13).

## Metabolic Syndrome in Adults With Opioid Use Disorder

Research has demonstrated that adults with OUD undergoing treatment had increased appetite and weight several months into the program (14, 15), with a 30% prevalence of metabolic syndrome, higher than the general population (16). Methadone exposure, compared to buprenorphine, was associated with worse metabolic syndrome in individuals with heroin use disorder (17). The rate of DM in patients on methadone was much higher than in the general population, while those on buprenorphine had a similar DM risk as the general population (18). This difference could be explained by the role of μ-opioid receptor (MOR) in the metabolic pathway, regulating fatty acid oxidation and promoting fat storage (19). Further, the κ-opioid receptor (KOR) is involved in hepatic lipid metabolism and signals fat storage (20). Therefore, methadone, a full MOR and a KOR agonist, exerts stronger metabolic side effects than buprenorphine, a partial MOR agonist and a KOR antagonist (21). Methadone also antagonizes the N-methyl-D-aspartate receptors, which normally function as an appetite suppressant (22). Despite this clinical evidence, it remains unclear whether these metabolic effects result from the medications or the lifestyle changes related to being in the program. Alternatively, obesity itself is associated with chronic pain and discomfort, leading to an increased use of opioids and other pain-controlling medications (23, 24).

The activation of reward circuitry seems to underlie the propensity for both food and drug ingestion. Increasing evidence shows that common genes implicated in addictive behaviors such as dopamine (*DA*), proopiomelanocortin (*POMC*), and leptin receptor (*LEPR*), may also be implicated in food addiction, particularly palatable sweet and fatty foods (12). Other genes involved in opioid consumption and appetite regulation include transcription factor 7 like 2, melanocortin-4, orexin-1, and opioid receptor μ-1 (25–28). Positron emission tomography (PET) imaging of individuals with obesity showed reductions in the striatal dopamine receptor type 2 (*DRD2*) that were proportional to body mass index (BMI), evidence that dopamine deficiency may induce pathological eating as a mechanism to offset the reward circuitry (10). Volkow *et al*. demonstrated that brain imaging of subjects with obesity had reduced striatal *DRD2* expression, similar in magnitude to reductions seen in subjects with cocaine addiction, further supporting a similar modulation of reward pathways in individuals with food and drug addictions (29–31).

While opioids induce signs of insulin resistance, metabolic changes, obesity, and diabetes, opioid-antagonists such as naltrexone reduce body weight and indices of metabolic syndrome (32). A clinical trial in patients with schizophrenia treated with olanzapine and randomized to naltrexone demonstrated a significant decrease in fat mass, an increase in fat-free mass, and a trend of improved insulin resistance compared to the placebo (32). These findings suggest that opioid antagonists effectively ameliorate the gain of body fat mass induced by olanzapine, an opioidergic compound. Additionally, an injection of long-acting depot naltrexone in opioid-dependent patients reduced self-reported hedonic and motivational characteristics of sucrose, which, in turn, was associated with diminished cue-induced opioid cravings (33). This study supports the link between opioid neurotransmission, hedonic signaling, and metabolic effects.

Other studies showed a less clear linkage between OUD and metabolic syndrome. A retrospective cross-sectional study of 10,032 subjects showed that drug use was linked to a higher waist circumference but did not find a significant association between drug use and clusters of three or more cardiometabolic disease risk factors (impaired glucose tolerance, insulin resistance, hypertension, dyslipidemia, and central adiposity) (9). Complicating this linkage is that adults with OUD have multiple life stressors and psychiatric comorbidities that may contribute to the development of metabolic syndrome (34, 35). Preclinical studies allow an opportunity to mechanistically understand the impact of opioid use on the development of metabolic syndrome in a more controlled setting.

## The Intersection of Food and Drug Addiction: Animal Data

Animal studies have shown that injections of morphine lead to hyperphagia and an increased intake of preferred foods (fat versus carbohydrates), resulting in weight gain. At the same time, naloxone, a MOR antagonist, decreased the intake of the preferred diet (36–39). The stimulation of MOR by injection of morphine into the lateral septum (LS) also reliably increased feeding behavior in a dose-responsive manner. In contrast, injection of high doses of MOR antagonist suppressed feeding (40). Another rodent experiment showed that administration of a highly selective MOR antagonist reduced food seeking *after* ingestion of palatable food, pointing to the reduction in the hedonic value of food. However, the MOR antagonist also reduced the seeking response *before* the delivery of palatable food. Therefore, this highly selective MOR antagonist reduced not only the hedonic/reward value of the highly palatable food but also the incentive motivational drivers that control food seeking (41), serving as evidence of direct effects of MOR on feeding behavior.

Animal research also showed that obesity, in turn, modulates the opioid system. Maternal consumption of high-fat diets and obesity was shown to upregulate the expression of *MOR* in offspring brain (42). Interestingly, there was an interaction between maternal obesity status and offspring sex, with pregestational obesity affecting male offspring only, while gestational obesity affecting both male and female offspring (42). Other studies also demonstrated that dams on high-fat diets produced offspring with a preference for high-fat diets, hyperphagia, and altered expression of *MOR* in male offspring (43). Furthermore, MOR is also susceptible to pre-pregnancy and gestational obesity, evidenced by reduced methylation in the promoter regions of MOR and the dopamine reuptake transporter, in addition to global DNA hypomethylation in the mouse brain. These behavioral and epigenetic effects were blocked with either the administration of MOR antagonist or methyl donor supplementation (44), mechanistic evidence of the intricate relationships between obesity, opioid receptor activation, and dysregulated feeding behavior.

## Neural and Systemic Inflammation of Opioids

**Figure 1:**
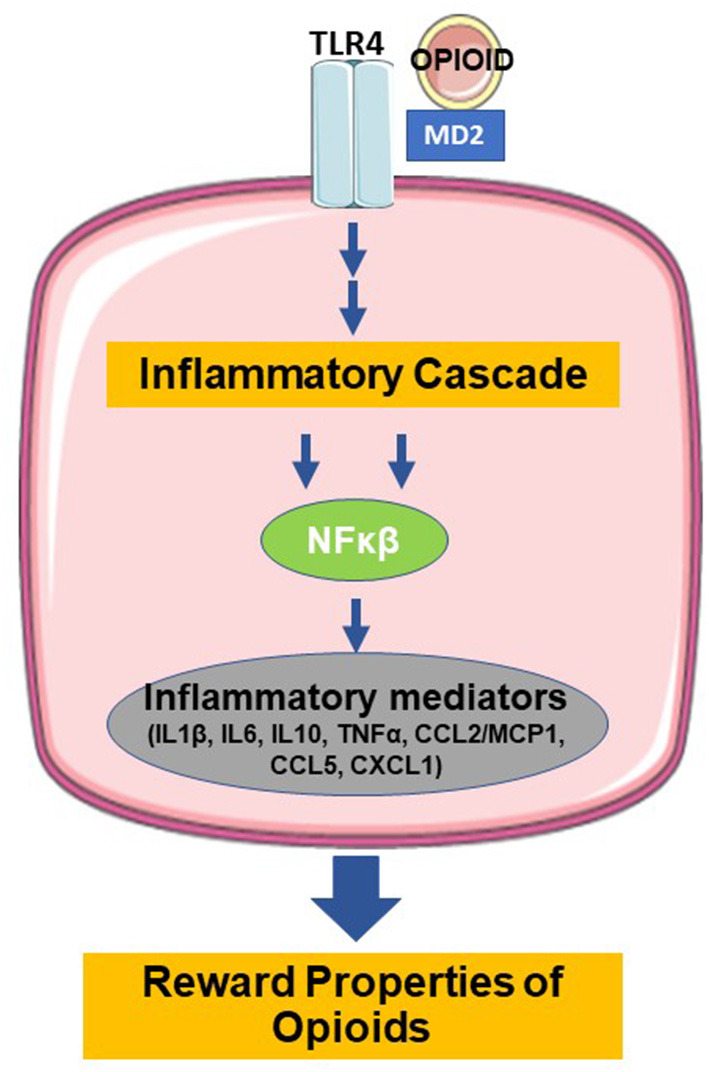
Binding of opioids with TLR4 induces the release of inflammatory mediators and modulation of the reward properties (TLR4=toll-like receptor type 4, MD=myeloid differentiation, NFκB=nuclear factor kappa beta, IL1β =interleukin 1 beta, IL6=interleukin 6, IL10=interleukin 10, TNFα =tumor necrosis factor alpha, CCL2=C-C motif chemokine ligand 2, MCP1=monocyte chemoattractant protein 1, CCL5=C-C motif chemokine ligand 5, CXCL1=C-X-C motif chemokine ligand 1. Figure created using derivatives of “Cellular Biology,” Servier Medical Art (https://smart.servier.com/) under the Creative Commons License Attribution 3.0 Unported License).

In addition to the direct opioid effects on opioid and feeding receptors, opioids also act via non-opioid receptors in the brain, particularly microglia, and induce neuroinflammation through their role as ‘foreign' agents. Microglia, which originate from yolk sac macrophages and migrate into the central nervous system (CNS) during embryogenesis, serve as the brain's innate immune system and regulators of CNS homeostasis (45, 46). Classical activation of microglia stimulates pattern recognition receptors that sense pathogen- and damage-associated molecular patterns in microorganisms, tumor cells, dying cells, or particles released in response to tissue injury and inflammation (47). Figure 1 shows that opioid binding to TLR4 on microglia activates nuclear factor-κB and the release of proinflammatory mediators (46, 48–51). TLR4 activation by opioids is similar to its classic ligand, lipopolysaccharides (52). The opioid-TLR4 signaling also induced extracellular DA increase in rodent NAc accompanied by reward-seeking behavior, e.g., conditioned place preference and opioid self-administration (53). This proinflammatory cascade is thought to actively oppose the analgesic effects of morphine and modulate the reward properties and the development of tolerance (54). Antagonism of TLR4 in animal models prevented the development of morphine tolerance in a dose-dependent manner, and injections of TLR4 agonists produced a novel tolerance to morphine (55). Together, these results confirmed the role of TLR4 in the regulation of morphine tolerance and showed that TLR4 might be a potential therapeutic target for analgesia management.

Jantzie *et al*. demonstrated that prenatal methadone elicits a systemic inflammatory response, leading to neuroinflammation, CNS injury, immune system dysfunction, and sustained peripheral immune hyperreactivity (56). Pups of dams implanted with mini-osmotic methadone pumps had significantly increased inflammatory cytokines in their peripheral blood mononuclear cells (PBMC) at P10, with IL1β significantly increased by P21, evidence of prolonged peripheral inflammation. Stimulation of PBMCs in the methadone-exposed pups with LPS resulted in the hypersecretion of cytokines compared to the saline-treated PBMCs, evidence of sustained immune hyperreactivity. Treatment with naloxone dissipated this cytokine response. The cortical section from methadone-exposed rats showed significantly elevated expression of *Tlr4, Myd88*, and key cytokines and chemokines, evidence of the opioid effects via the TLR4-dependent pathway. Furthermore, biochemical assessment and diffusion tensor imaging of the brain showed decreased myelin maturity and disrupted microstructural brain integrity in the methadone cohort, with executive function and cognitive impairment into adulthood. Prenatal opioid exposure, therefore, adversely modulates systemic inflammatory response accompanied by molecular, structural, and behavioral/cognitive consequences (56).

Neuroinflammation is associated with obesity-driven disorders through the neuroendocrine effects of opioids (57). Animal models and human conditions of stress and depression showed that the neuroimmune impact of opioids dysregulates the hypothalamic-pituitary-adrenal axis and induces the release of peptides and hormones including cortisol. These glucocorticoids, in turn, regulate the expression of inflammatory cytokines in the hypothalamus, hippocampus, and prefrontal cortex (58). Prolonged neuroinflammation may lead to imbalanced hypothalamic hormonal signaling and subsequent dysregulated energy homeostasis, impaired control of food intake, development of insulin resistance, obesity, and cardiovascular disease (59–62).

**Figure 2:**
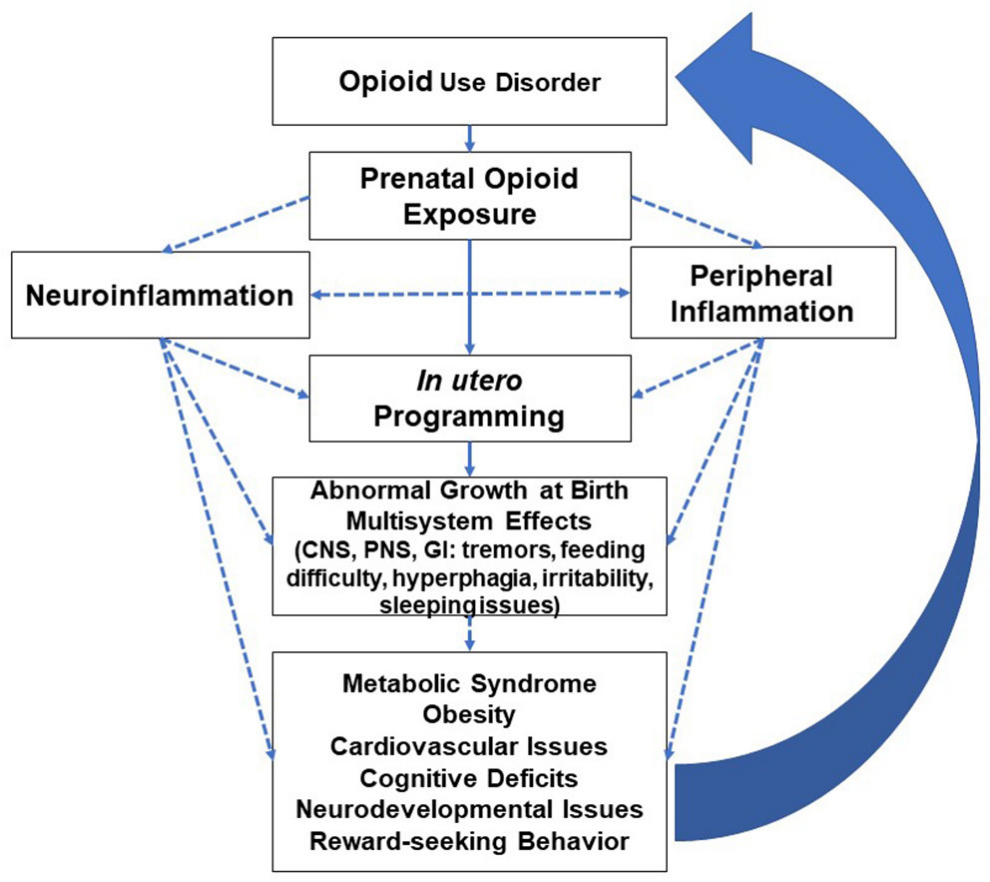
Inflammatory modulation of opioids and multiorgan effects with long-term adverse effects leading to increased predisposition to opioid use disorder (CNS=central nervous system, PNS=peripheral nervous system, GI=gastrointestinal system). Well-studied or known topics are indicated by solid lines; hypothetical topics or knowledge gaps are indicated by dashed lines.

While neuroinflammation disrupts insulin sensitivity and leptin resistance (63), obesity reciprocally impairs microglia function (64). Central and peripheral inflammation processes are in constant communication and create feedback signaling that predisposes opioid-exposed individuals to adverse neurological and peripheral effects, i.e., cognitive and cardiometabolic deficits (65, 66). Obesity as a pro-inflammatory state disrupts the blood-brain barrier and reduces the expression of tight junction proteins, enhancing the permeability and recruitment of peripheral/plasma constituents with the resultant microglial inflammatory activation (66–68). Neonates with an immature blood-brain barrier are at a higher risk for both peripheral and neurological effects of prenatal opioids and are prone to the vicious cycle depicted in Figure 2.

## Growth Dysregulation in Opioid-Exposed Neonates and Adverse Metabolic Risks

While studies increasingly found adverse neurodevelopmental outcomes and poor academic performance in children exposed to opioids *in utero* (69–71), the somatic growth trajectory of these neonates is poorly understood. *In utero* exposure to opioids and other illicit drugs is linked to prematurity, and smaller overall size, head circumference, and brain volume at birth (72–76), all postulated to be multifactorial, i.e., a poor diet during pregnancy, compromised placental transfer of nutrition, and direct effects of the drugs on fetal growth (77, 78). Exposed neonates have lower growth velocity that persists to 10 years of age (79–81). Others have reported that despite the lower growth parameters at one year, neonates demonstrate catch-up growth starting at 4 months, with a more prominent catch-up in weight by 12 months of age (82). Evidence of catch-up growth has also been reported by Shankaran *et al*., who reported growth discrepancies disappearing by 2 years of age (for weight and head circumference) and by 3 years of age (for height) (83).

A longitudinal study of 238 children with prenatal cocaine exposure and 323 children without exposure showed a four-fold obesity risk in cocaine-exposed children at 9 years of age (84). These exposed neonates were significantly small for gestational age (SGA) and had more rapid earlier weight gain. When analyzed based on prenatal cocaine-by-alcohol exposure, there was no difference in the prevalence of obesity from birth to 6 years of age. However, by age 7, children who were exposed to cocaine but not alcohol were more obese than those exposed to both substances, to neither substance, or to alcohol but not cocaine. This pattern continued to 9 years of age. Similarly, children with prenatal cocaine but not alcohol exposure had higher BMI Z-scores than those not exposed to either substance, with a difference in the Z-scores noted between 3 and 9 years of age, but not earlier. Obesity was associated with faster early weight gain, high caloric intake, and inadequate exercise. However, a key predictor of obesity at 9 years of age is the rate at which neonates grow from birth to one year, supporting that rapid catch-up growth in the first year of life proved to be most impactful on metabolism later in life (85, 86). A similar phenomenon has been observed in neonates with intrauterine growth restriction. The *in utero* nutritional deficiency compromises fetal growth and organ differentiation. quickly replaced by ample postnatal nutritional supply and a period of rapid catch-up growth. The unique fetal programming and aberrant postnatal growth trajectory predispose these neonates to a higher incidence of metabolic syndrome, early signs of atherosclerotic diseases, and increased cardiovascular risks (87, 88).

A pilot study looking at air displacement plethysmography measurements in neonates with NAS showed a rapid increase in the mean body mass, fat-free mass, and fat percentage by 4 months of life (89). This study showed that these neonates were born smaller and remained leaner in the first few weeks of life. However, by 4 months of age, neonates with NAS had similar mean body mass and fat-free mass measurements as the non-exposed neonates, with doubling of mean fat mass percentage from 14% to 28% between weeks 4 and 16. The rapid increase in the body mass of neonates with NAS may predispose them to future obesity. Compared to non-exposed neonates, those with NAS also had a larger variability in growth in the first year of life compared to non-exposed neonates (90), evidence of a distinct growth pattern that may place them at risk for subsequent aberrant development.

## Molecular and Behavioral Evidence of Sex-Specific Impact of Prenatal Opioids on Neonatal Feeding Regulation and Future Implications

In addition to the abnormal growth parameters described above, opioid-exposed neonates exhibit a unique feeding phenotype that may compound their future metabolic risk. An early withdrawal sign in NAS is poor oral coordination shortly after birth, followed by excessive eating/hyperphagia thereafter (91–93). While most neonates are discharged home without feeding difficulty, many continue to exhibit hyperphagia, consuming up to two-fold the caloric intake of healthy newborns. Using neonatal saliva, our laboratory studied the expression of select hypothalamic and reward genes that regulate feeding—neuropeptide Y2 receptor (*NPY2R*), *POMC, LEPR, DRD2* (13). A higher proportion of opioid-exposed males required pharmacotherapy (41% males vs. 22% females). While there was no significant difference in the gene expression between opioid- and non-exposed neonates, stratification by sex showed that opioid-exposed males had significantly higher expression of *DRD2* than females. Furthermore, males who required pharmacotherapy had significantly higher expression of *DRD2* and *LEPR* than females, evidence that prenatal opioid exposure creates an imbalance between reward (*DRD2*) and anorexigenic (*LEPR*) signaling. The expression of *DRD2* also correlated significantly with volume intake, evidence that hyperphagia may be a compensatory behavior to replace the reward signaling provided *in utero*. Males with pharmacotherapy requirement consumed an average of 180 mL/kg/day by one week of life and 210 mL/kg/day on the day of discharge, 15% above the amount ingested by females.

The expression of *DRD2* in neonates requiring pharmacotherapy also showed a trend of positive correlation with percent weight change at discharge, evidence of early catch-up growth seen in other studies. Our study supports the hypothesis that opioid-exposed neonates exhibit hyperphagia early in life that continues throughout the withdrawal course, with males displaying more molecular and behavioral effects than females. While limited by the small number of subjects and duration (hospital stay), our study provided the foundation for an ongoing study examining the sex-specific impact of prenatal opioid exposure in a larger cohort and beyond the hospital stay. The higher expression of *DRD2* may carry important implications for future reward-seeking behavior, albeit drugs or food. Sex-specific findings in our study may explain the predominance of males in OUD (94, 95) and male sex as a risk factor for more severe NAS and pharmacotherapy requirement (96). Additionally, serial salivary *DRD2* may predict if opioid-exposed neonates are at risk for an early hyperdopaminergic followed by a hypodopaminergic state, e.g., through a desensitizing process over time. Subjects with chronic OUD and obesity showed evidence of dopamine deficiency that may induce compensatory and pathological behaviors exacerbating their drug or food dependency (30–33).

The higher expression of *LEPR* in our opioid-exposed male cohort may induce a hyperleptinemia state that triggers insulin resistance and cardiometabolic complications. While leptin serves as satiety signaling in the context of energy surplus (97), chronic hyperleptinemia may lead to blunted responses and obesity (97). A study in adults undergoing weight-loss treatment programs showed associations between food craving, overeating, and genetic polymorphisms involved in addiction, as well as a significant gene-gene interaction between *DRD2* and *LEPR*, which synergistically influences the development of severe obesity (98). Our data highlighted the sex-specific impact of prenatal opioid exposure on the developing brain accompanied by anthropometric and behavioral changes, predisposing these neonates to future reward-seeking behaviors with adverse growth, neurodevelopmental, and cardiometabolic consequences. Given the intersection between food and drug dependence, future studies ought to understand the metabolic consequences of higher reward signaling, abnormal feeding phenotype, and rapid catch-up growth in these neonates.

## Conclusion

Animal and human studies highlight the intricate and complex intersection between reward and feeding receptors in the brain, as well as central and peripheral inflammation that may explain the linkage between OUD and metabolic syndrome. Emerging studies in the neonatal population, including ours, indicate that the adverse effects of prenatal opioids start early on in life. However, these topics are understudied and remain significant knowledge gaps in the neonatal and pediatric population, either due to the fragile nature of the population or the limited non-invasive and validated methodologies available to this group. Additionally, the socioeconomic circumstances encountered by families with OUD pose unique challenges that may hinder robust long-term follow-up, further limiting comprehensive understanding of the cardiometabolic impact of prenatal opioids. Despite these obstacles, the field urgently needs robust research to understand the impact of prenatal opioids on brain reward signaling, inflammatory modulation of opioids, hyperphagia, and abnormal growth, including rapid catch-up growth in these neonates, and long-term cardiometabolic consequences. Animal and human adult data on the inflammatory impact of opioids provide an opportunity to develop and utilize non-opioid interventions, such as anti-inflammatory agents, in dealing with postnatal withdrawal signs and future metabolic syndrome. Furthermore, research in these topics will enable non-pharmacological interventions, such as nutritional programs and cognitive behavioral therapy, that could potentially ameliorate inflammation and prevent the development of metabolic syndrome in opioid-exposed infants and children. Well-funded public health research will enable precision and personalized medicine, such as more individualized and balanced nutrition to reduce the likelihood of early rapid catch-up growth, diligent monitoring of nutritional intake over time, exercise regimen and cognitive behavioral interventions that reduce the neuroinflammatory impact of opioids on future neurodevelopment and somatic growth, personalized health surveillance, e.g., early lipid screening, all of which may halt the vicious cycle that predisposes these growing neonates to adverse health outcomes in adulthood.

## Author Contributions

EY: paper concept, writing and editing of manuscript, figure concept and creation. JM: paper concept and manuscript editing. All authors contributed to the article and approved the submitted version.

## Conflict of Interest

The authors declare that the research was conducted in the absence of any commercial or financial relationships that could be construed as a potential conflict of interest.

## Publisher's Note

All claims expressed in this article are solely those of the authors and do not necessarily represent those of their affiliated organizations, or those of the publisher, the editors and the reviewers. Any product that may be evaluated in this article, or claim that may be made by its manufacturer, is not guaranteed or endorsed by the publisher.
